# Supplementary material for: The Cep57-pericentrin module organizes PCM expansion and centriole engagement
Source: Nat Commun. 2019 Feb 25;10:931. doi: 10.1038/s41467-019-08862-2 (PMC6389942; doi:10.1038/s41467-019-08862-2)
Supplement: Supplementary file 1 — Supplementary Information [file 41467_2019_8862_MOESM1_ESM.pdf]

Supplementary information

The Cep57-pericentrin module organizes PCM expansion and centriole engagement

Watanabe *et al.*

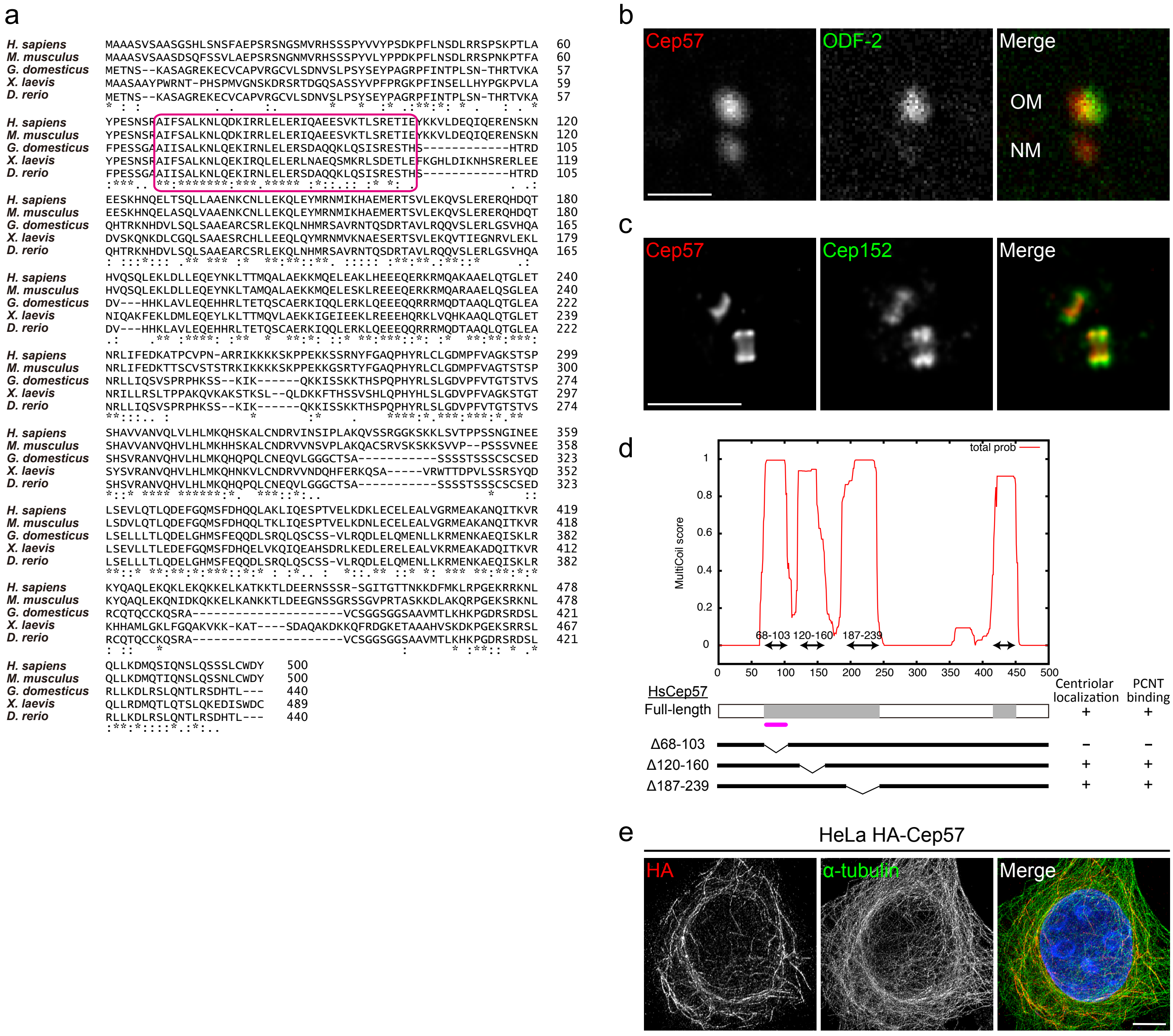

Supplementary Figure 1. Cep57 is a conserved mother centriolar protein

(**a**) Alignments of full-length Cep57 within *H. sapiens*, *M. musculus*, *G. domesticus*, *X. laevis*, *D. rerio*. Note that full-length Cep57 is evolutionarily conserved in vertebrates. (**b**) The difference in Cep57 signal intensity between old mother and new mother centrioles in interphase. HeLa cells were immunostained with antibodies against Cep57 (red) and ODF-2 (green, old mother centriole marker). Cep57 signal intensity at old mother centrioles (OM, ODF-2 positive) was higher than that of new mother centrioles (NM, ODF-2 negative). Scale bar, 1 µm. (**c**) The difference in Cep57 signal intensity between OM and NM centrioles was derived from the height of Cep57 that is located on the centriole wall. HeLa cells were immunostained with antibodies against Cep57 (red) and Cep152 (green). Scale bar, 1 µm. (**d**) Secondary structure prediction of Cep57. The analysis shows that there are three coiled-coil motifs in the first coiled-coil domain. Schematic of Cep57 full-length and the deletion mutants that lack each coiled-coil motif. The right column shows a summary of centriolar localization of the Cep57 constructs examined in HeLa cells. (**e**) Overexpressed Cep57 localized to microtubules. HeLa cells expressing HA-Cep57 were immunostained with antibodies against HA (red) and α-Tubulin (green). Scale bar, 5 µm.

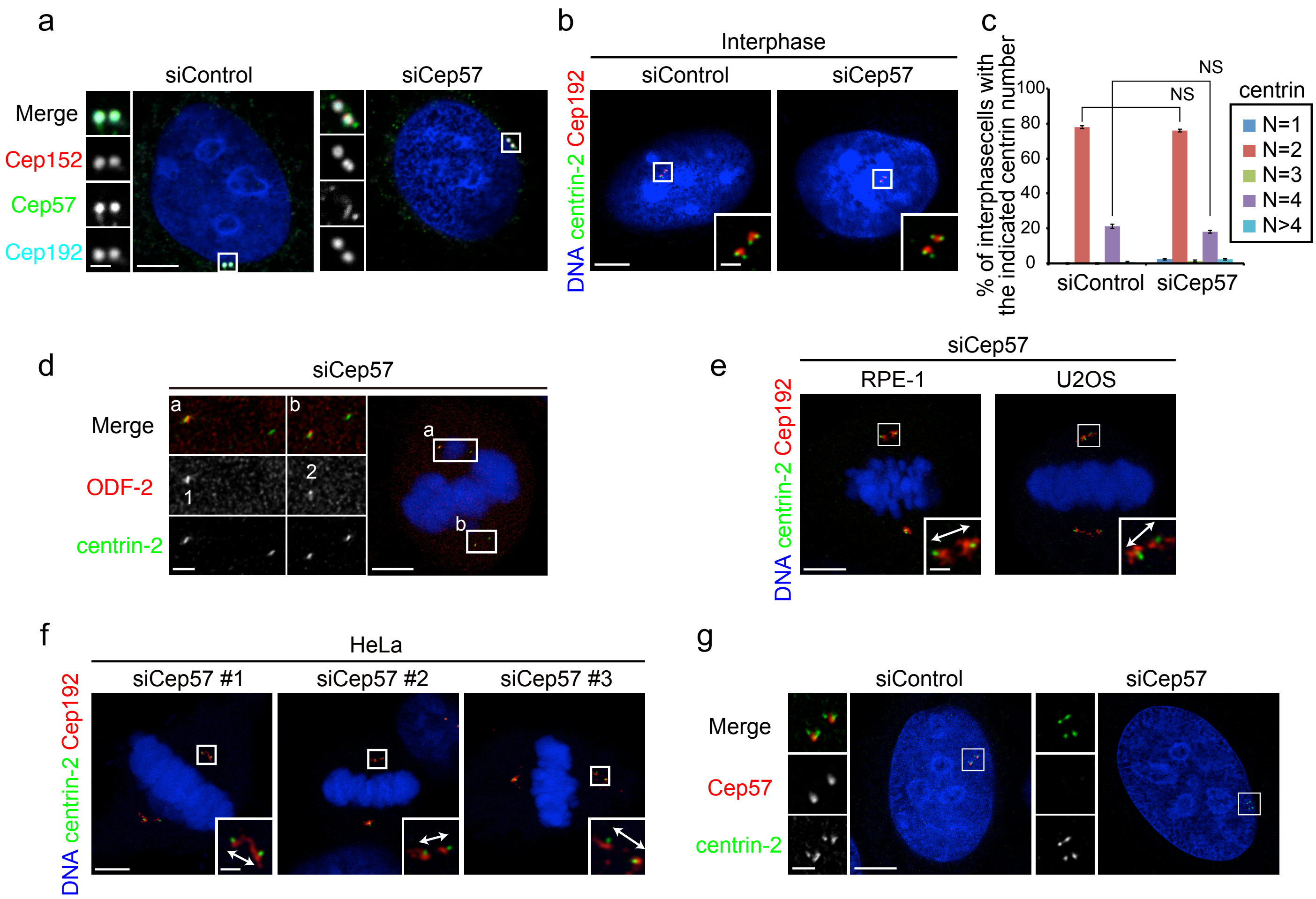

Supplementary Figure 2. Effect of Cep57 depletion in interphase cells and efficiency of Cep57 siRNAs

(**a**) Depletion of Cep57 did not affect the centriolar loading of Cep152. HeLa cells were treated with siControl or siCep57 and immunostained with antibodies against Cep57 (green), Cep152 (red) and Cep192 (cyan). (**b**) Depletion of Cep57 did not lead to significant defects in PCM formation or centriole duplication in interphase. HeLa cells were treated with siControl or siCep57. and immunostained with antibodies against centrin-2 (green) and Cep192 (red). (**c**) Histograms represent frequency of interphase cells with the indicated centrin number. Values are mean percentages ± s.d. from three independent experiments (*N*=100 for each experiment). Two-tailed, unpaired Student's t-test was used to obtain *P* value. *, $p < 0.05$; **, $p < 0.01$; NS, not significantly different ($p > 0.05$). (**d**) The two centrioles seen at the pole are a pair of mother-daughter centrioles. HeLa cells were treated with siControl or siCep57 and immunostained with antibodies against centrin-2 (green) and ODF-2 (red). (**e**) RPE-1 and U2OS also exhibited precocious centriole disengagement and PCM disorganization upon Cep57 depletion. The cells were immunostained as in (b). (**f**) HeLa cells were treated with siRNA targeting against different sequences of Cep57. The cells were immunostained as in (b). (**g**) The efficiency of siRNA targeting Cep57 and the specificity of Cep57 antibody were confirmed by immunofluorescence analysis. HeLa cells were treated with siCep57 and were immunostained with antibodies against centrin-2 (green) and Cep57 (red). All scale bars, 5 μm in the low-magnified view, 1 μm in the inset. Left-right arrows indicate precociously-disengaged centrioles.

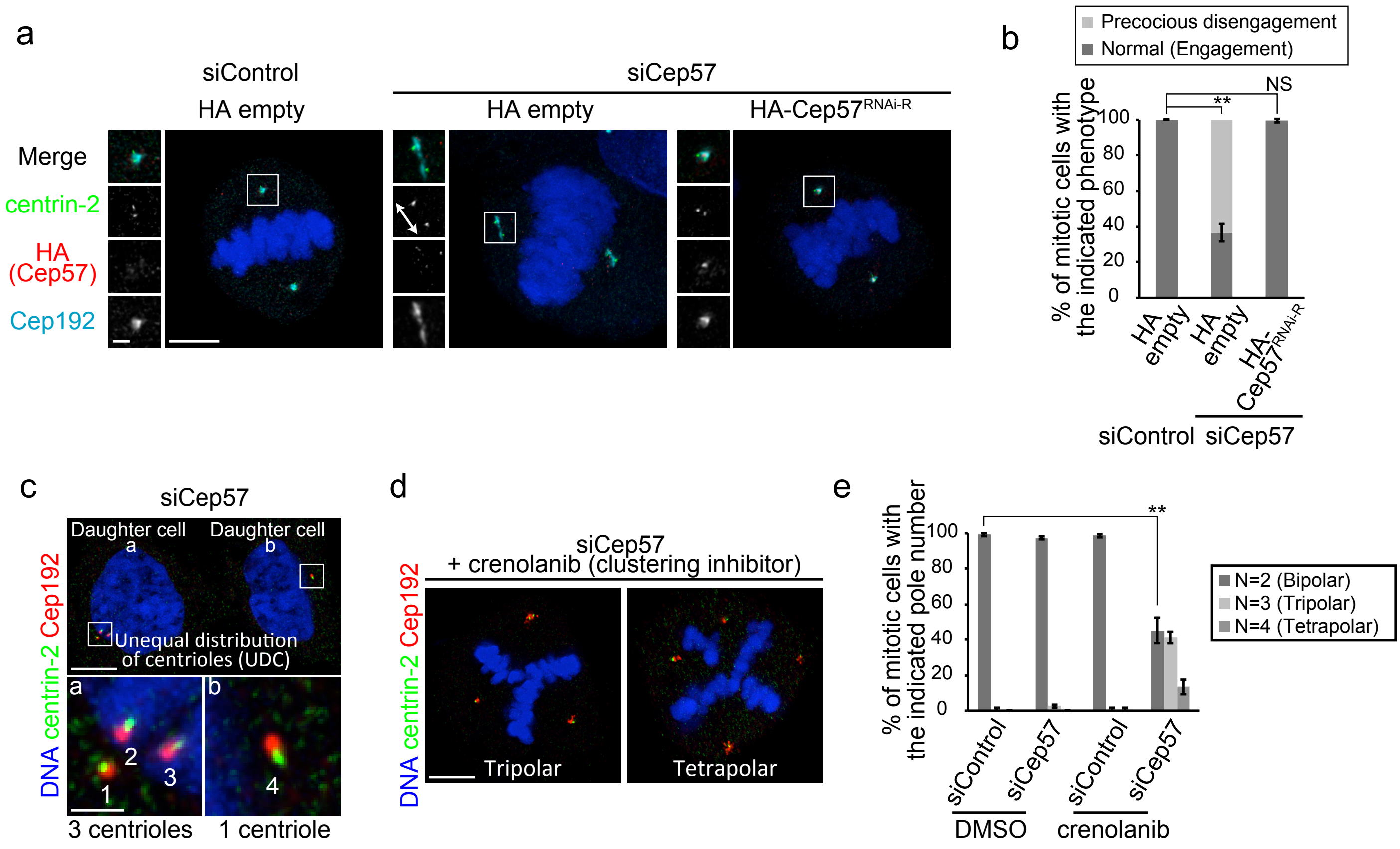

Supplementary Figure 3. Cep57 rescue experiments and effect of crenolanib (clustering inhibitor) in Cep57-depleted cells

(**a**) Expression of RNAi-resistant (RNAi-R) form of Cep57 restored the phenotype. HeLa cells were treated with siControl or siCep57, followed by transfection with HA empty (control) or RNAi-resistant HA-Cep57. The cells were immunostained with antibodies against centrin-2 (green), HA (red) and Cep192 (cyan). (**b**) Histograms represent frequency of mitotic cells with the indicated phenotypes in (a). Values are mean percentages ± s.d. from three independent experiments (*N*=50 for each experiment). (**c**) Unequal distribution of centrioles results in two daughter cells with three centrioles and one centriole, respectively. HeLa cells were treated with siControl or siCep57. and immunostained with antibodies against centrin-2 (green) and Cep192 (red). (**d**) Crenolanib treatment increased the number of mitotic cells with multipolar spindles in Cep57-depleted cells. HeLa cells were treated with siControl or siCep57, followed by treatment with DMSO or crenolanib (500 nM) for 6 h. The cells were immunostained as in (c). (**e**) Histograms represent frequency of mitotic cells with the indicated spindle pole number in (d). Values are mean percentages ± s.d. from three independent experiments (*N*=50 for each experiment). All scale bars, 5 μm in the low-magnified view, 1 μm in the inset. Left-right arrows indicate precociously-disengaged centrioles. Tukey's multiple comparisons test was used in (b) and (e) to obtain *P* value. *, *p* < 0.05; **, *p* <0.01; NS, not significantly different (*p* > 0.05).

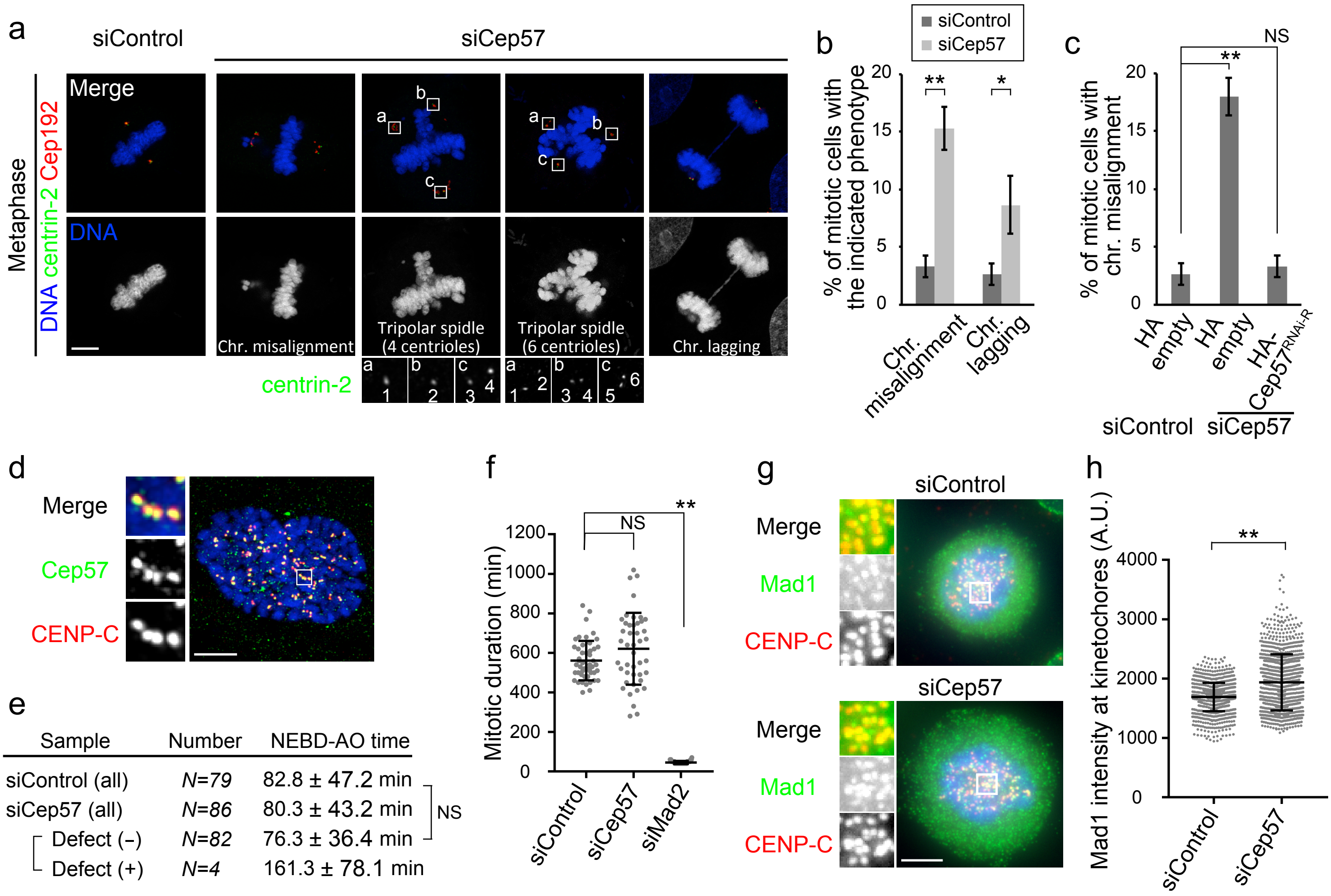

Supplementary Figure 4. Chromosomal segregation errors caused by Cep57 depletion are due to precocious centriole disengagement, rather than reduced SAC signaling

(**a**) Chromosome segregation errors observed in Cep57-depleted fixed cells. HeLa cells were treated with siControl or siCep57 and immunostained with antibodies against centrin-2 (green) and Cep192 (red). (**b**) Histograms represent frequency of mitotic cells with the indicated phenotypes observed in (a). Values are mean percentages ± s.d. from three independent experiments (*N*=50 for each experiment). (**c**) Chromosome segregation errors provoked by Cep57 depletion were rescued by expression of RNAi-resistant form of HA-Cep57. Histograms represent frequency of mitotic cells with chromosome misalignment. Values are mean percentages ± s.d. from three independent experiments (*N*=50 for each experiment). (**d**) The kinetochore signal of Cep57 could be detected in HeLa cells fixed with methanol/paraformaldehyde. HeLa cells were fixed with cold methanol for 7 min and 4% paraformaldehyde for 10 min. The cells were immunostained with antibodies against Cep57 (green) and CENP-C (red, kinetochore marker). (**e**) Table of the time from NEBD to AO in Fig. 3c. Cep57-depeleted cells were classified based on the presence of chromosome misalignment, because of ectopic MTOCs including multipolar spindle formation (defect (-/+)). The defect-positive cells with ectopic MTOCs tend to exhibit a mitotic delay. (**f**) Quantification of mitotic duration of HeLa cells with nocodazole treatment. HeLa cells were treated with siControl, siCep57, or siMad2, followed by treatment with nocodazole (100 nM) for 1 h. After nocodazole treatment, the cells were visualized for live imaging. (**g**) Depletion of Cep57 did not affect kinetochore localization of Mad1. HeLa cells were treated with siControl or siCep57, followed by treatment with nocodazole (100 nM) and MG132 (5 μm) for 1 hour. The cells were immunostained with antibodies against Mad1 (green) and CENP-C (red). (**h**) Dot plots represent quantification of the signal intensity of Mad1 at kinetochores. Values are mean intensity ± s.d. (N>1000) All scale bars, 5 μm. Two-tailed, unpaired Student's t-test was used in (b) and (h) to obtain *P* value. Tukey's multiple comparisons test was used in (c), (e) and (f) to obtain *P* value. *, $p < 0.05$; **, $p < 0.01$; NS, not significantly different ($p > 0.05$).

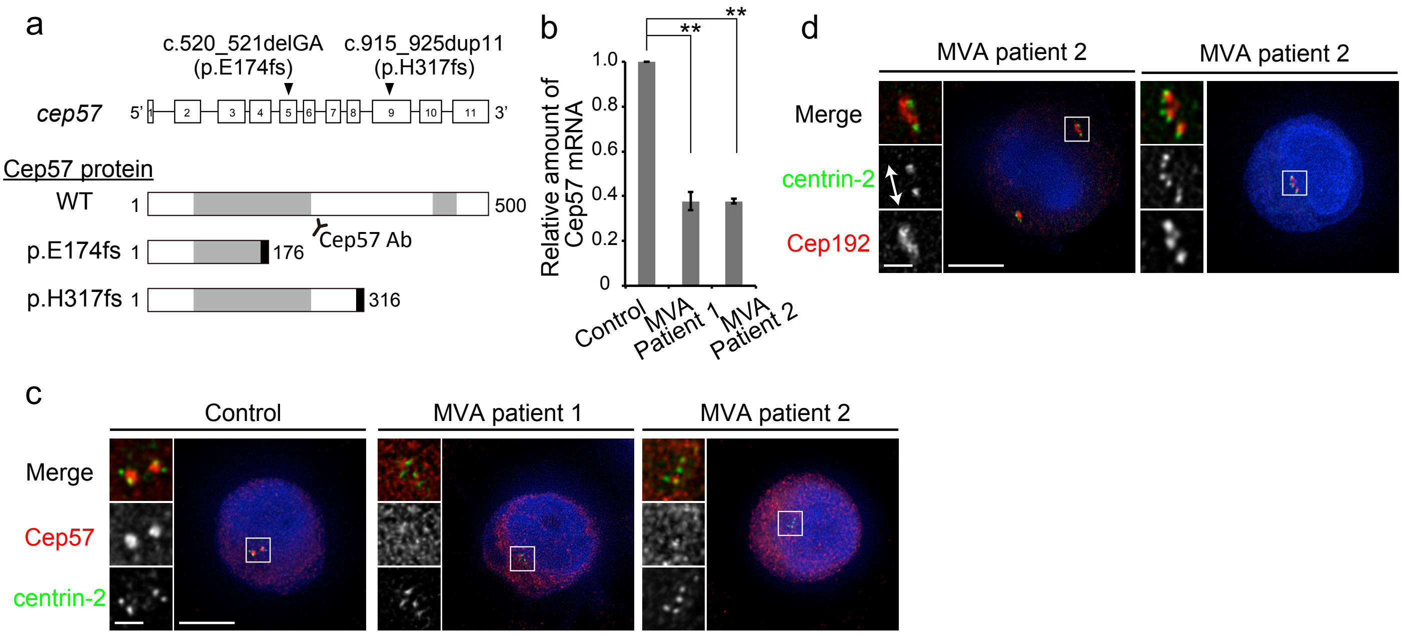

Supplementary Figure 5. Characterization of the MVA patients' cells

(**a**) Schematic representation of the genomic structure of *cep57*. Arrowheads indicate the positions of mutations. Schematic representation below shows the protein structure of full-length or truncate mutants of Cep57. Gray and black boxes indicate coiled-coil motifs and frameshifted regions, respectively. (**b**) Quantitative PCR analysis of Cep57 mRNA amount in the MVA patients' cells. Values are mean percentages ± s.d. from three independent experiments. Dunnett's multiple comparisons test was used to obtain *P* value. *, $p < 0.05$; **, $p < 0.01$; NS, not significantly different ($p > 0.05$). (**c**) The signal of Cep57 was not detectable at centrosomes in the MVA patients' cells. MVA patients' LCLs and their mother's LCL (unaffected control) were immunostained with antibodies against centrin-2 (green) and Cep57 (red). (**d**) MVA patient 2 cells also exhibit precocious centriole disengagement (left-right arrow, left panel) and abnormalities in centrosome number (right panel) as is the case of MVA patient 1 cells. All scale bars, 5 μm in the low-magnified view, 1 μm in the inset. Left-right arrows indicate precociously-disengaged centrioles.

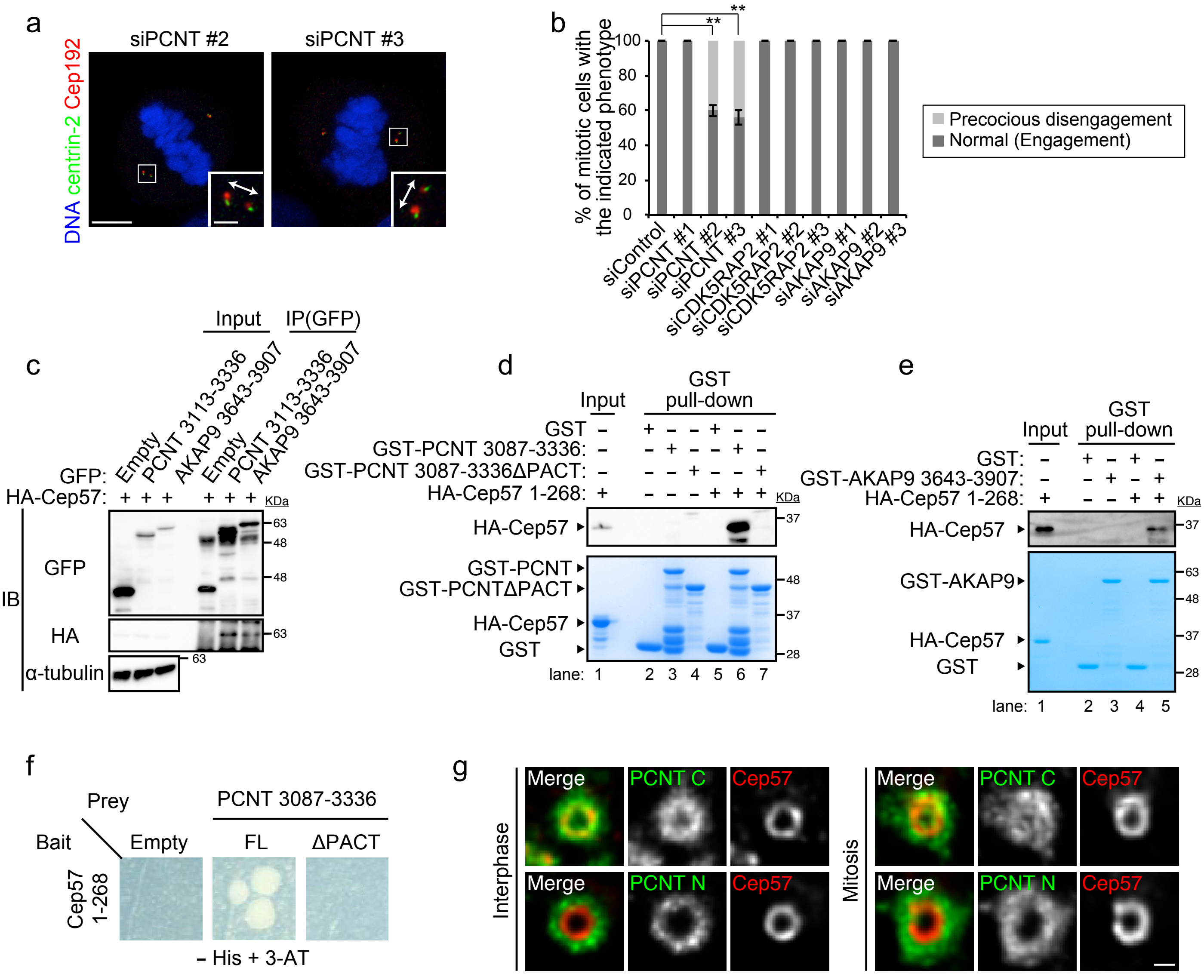

Supplementary Figure 6. The Cep57-PCNT interaction and co-localization

(**a**) Depletion of PCNT caused precocious centriole disengagement whereas depletion of CDK5RAP2 and AKAP9 did not affect centriole engagement in mitosis. Left-right arrows indicate precociously-disengaged centrioles. Scale bar, 5 μm in the low-magnified view, 1 μm in the inset. (**b**) Histograms represent frequency of mitotic cells with the indicated phenotypes in each condition. Values are mean percentages ± s.d. from three independent experiments (N=50 for each experiment). Tukey's multiple comparisons test was used to obtain P value. *, $p < 0.05$; **, $p < 0.01$; NS, not significantly different ($p > 0.05$). (**c**) HEK293T cells co-expressing HA-Cep57 and GFP-PCNT or GFP-AKAP9 fragments containing the PACT domain were IPed with GFP antibodies. (**d**) GST pull-down assay showing the interaction between Cep57 and PCNT fragments *in vitro*. These bacterially purified recombinant proteins contain the interaction regions that were identified by the co-IP experiments in Fig. 4. Inputs (lane 1 for Coomassie blue staining, 66.7%; lane 1 for western blotting, 1/40000 volume of lane 1 in the Coomassie blue staining) and affinity-purified protein complexes (lane 2-7) were subjected to SDS–PAGE, stained (Coomassie blue staining), and analyzed by western blotting. (**e**) GST pull-down assay showing the interaction between Cep57 and AKAP9 fragments *in vitro*. These bacterially purified recombinant proteins contain the interaction regions that were identified by the co-IP experiments in Fig. 4. Inputs (lane 1 for Coomassie blue staining, 66.7%; lane 1 for western blotting, 1/30000 volume of lane 1 in the Coomassie blue staining) and affinity-purified protein complexes (lane 2-5) were analyzed as in (d) (**f**) Yeast two-hybrid assay showing the interaction between Cep57 and PCNT. The indicated clones were grown on the plates lacking histidine and containing 20 mM 3-amino-1,2,4-triazole (3-AT) at 30°C. The same results were obtained from two

independent clones for each combination. (**g**) STED images representing top views of Cep57 and PCNT (the N- or C-terminus) at mother centrioles. HeLa cells were immunostained with the indicated antibodies. Note that Cep57 co-localized with the C-terminus of PCNT, but not with the N-terminus of PCNT. Scale bar, 200 nm.

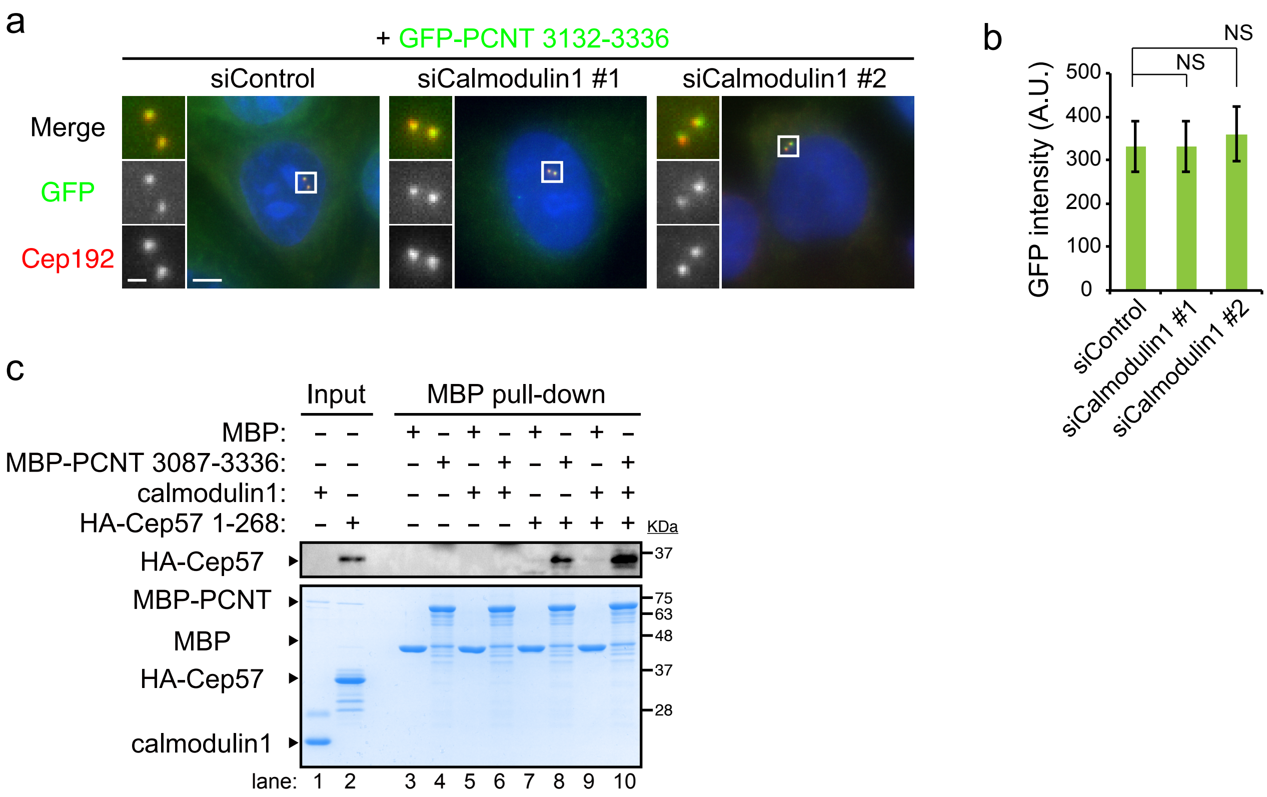

Supplementary Figure 7. Effect of calmodulin1 on centriolar PCNT loading and the Cep57-PCNT interaction

(**a**) HeLa cells were treated with siControl or siCalmodulin1, followed by transfection with the GFP-PCNT fragment containing the PACT domain (GFP-PCNT 3132-3226). The cells were immunostained with antibodies against GFP (green), Cep192 (red). Note that depletion of calmodulin1 did not affect PCM integrity and the centriolar loading of GFP-PCNT fragment containing the PACT domain in human cells (**b**) Histograms represent quantification of the signal intensity of GFP at old mother centrioles in (a). Values are mean intensities ± s.d. (siControl *N*=32, siCalmodulin1 #1 *N*=33, siCalmodulin1 #2 *N*=23). (**c**) Effect of calmodulin1 on the *in vitro* Cep57-PCNT interaction. MBP pull-down assay in Fig. 4f was performed with calmodulin1. Inputs (lane 1,2 for Coomassie blue staining, 25%; lane 2 for western blotting, 1/30000 volume of lane 2 in the Coomassie blue staining) and affinity-purified protein complexes (lane 3-10) were subjected to SDS–PAGE, stained (Coomassie blue staining), and analyzed by western blotting.

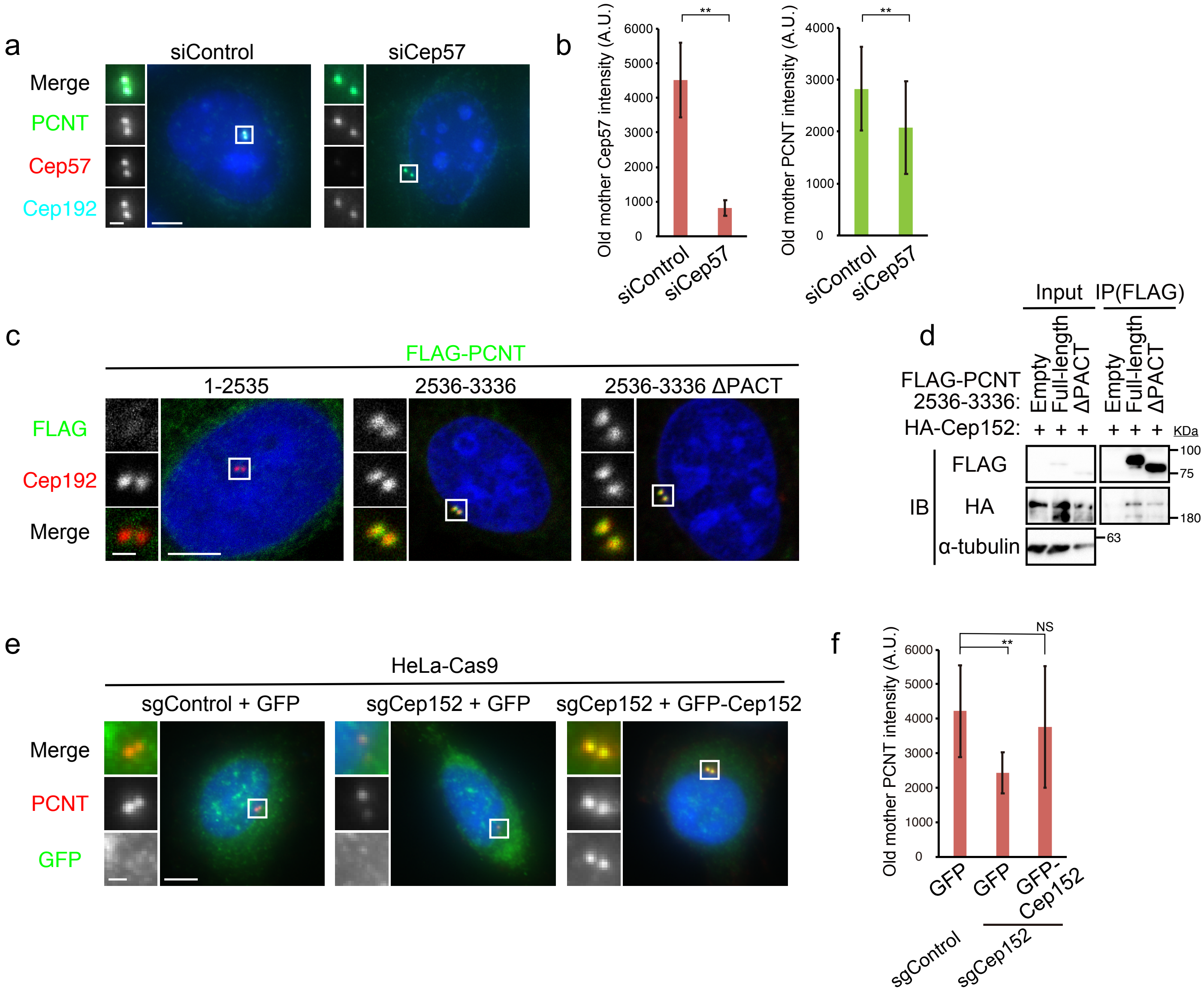

Supplementary Figure 8. Cep152 and Cep57 redundantly regulate centriolar PCNT loading in interphase cells

(**a**) HeLa cells were treated with siCep57. The cells were immunostained with antibodies against PCNT (green), Cep57 (red) and Cep192 (cyan). (**b**) Histograms represent quantification of the signal intensity of PCNT and Cep152 at old mother centrioles in interphase in (a) (siControl *N*=31, siCep57 *N*=31). Two-tailed, unpaired Student's t-test was used to obtain *P* value. (**c**) The C-terminus of PCNT has another centrosomal targeting domain other than the PACT domain. HeLa cells expressing the indicated FLAG-PCNT mutants were immunostained with antibodies against FLAG (green), Cep192 (red). (**d**) HEK293T cells co-expressing HA-Cep152 and FLAG-PCNT or the indicated mutants were IPed with FLAG antibodies. (**e**) HeLa cells stably expressing Cas9 were treated with control sgRNA (sgControl) or sgRNA targeting Cep152 (sgCep152), followed by transfection with GFP empty (control) or GFP-Cep152. The cells were immunostained with antibodies against GFP (green), PCNT (red). (**f**) Histograms represent quantification of the signal intensity of PCNT at old mother centrioles in interphase in (e) (*N*=20 for each condition). Tukey's multiple comparisons test was used to obtain *P* value. *, $p < 0.05$; **, $p < 0.01$; NS, not significantly different ($p > 0.05$). All scale bars, 5 μm in the low-magnified view, 1 μm in the inset.

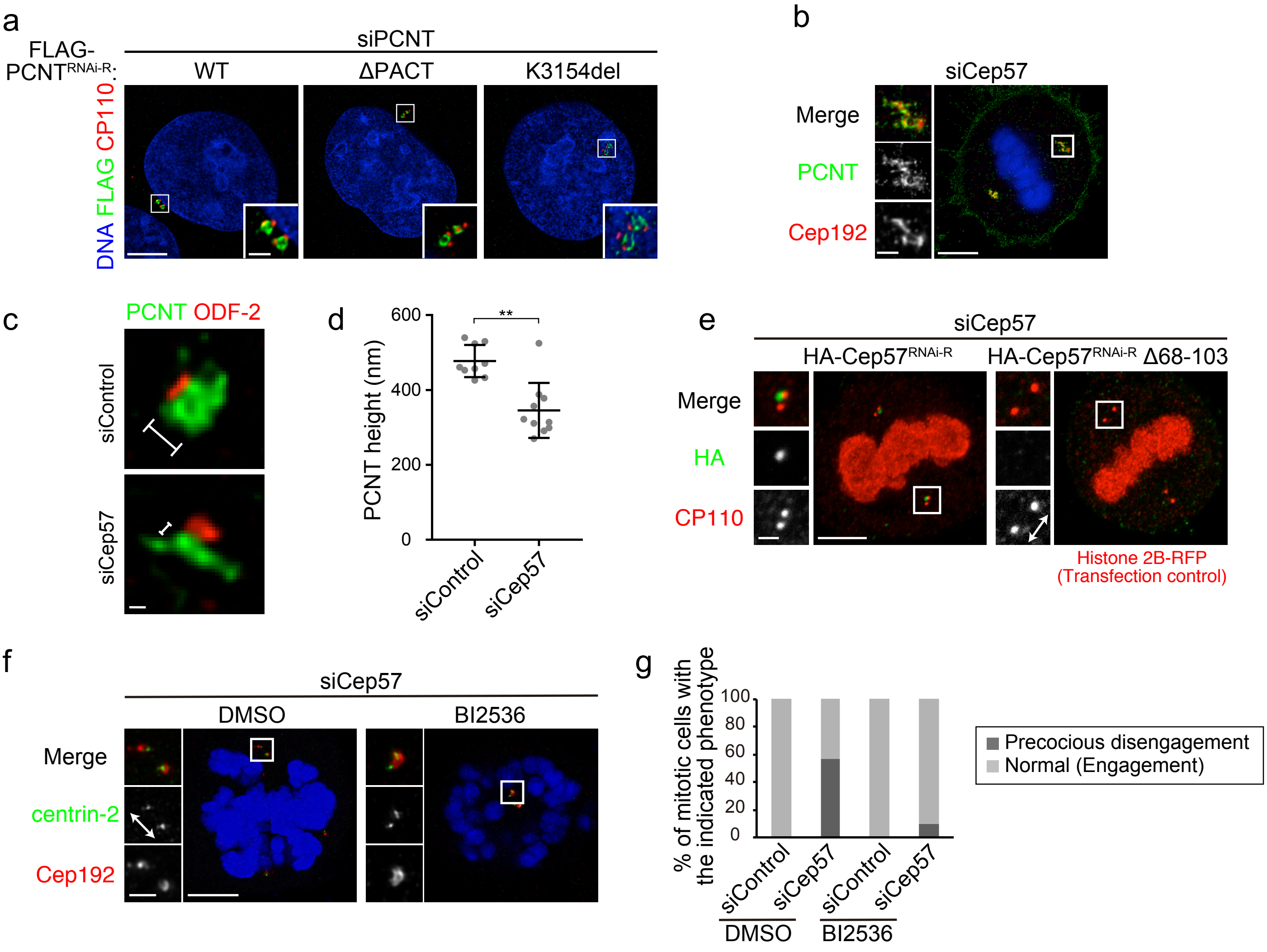

Supplementary Figure 9. Depletion of Cep57 causes the dispersion of PCNT and shortens the height of PCNT that binds along the side of centriolar wall

(**a**) Cep57-binding-deficient PCNT mutants (PCNT ΔPACT or K3154del) were normally recruited to the centrosome in interphase. HeLa cells were treated with siPCNT, followed by transfection with RNAi-resistant PCNT or the indicated mutants. The cells were immunostained with antibodies against FLAG (green), Cep192 (cyan) and CP110 (red). Scale bar, 5 μm in the low-magnified view, 1 μm in the inset. (**b**) Depletion of Cep57 caused the dispersion of PCNT. HeLa cells were treated with siCep57 and immunostained with antibodies against PCNT (green), Cep192 (red). Scale bar, 5 μm in the low-magnified view, 1 μm in the inset. (**c**) Images representing side views of PCNT associated with the centriole wall in mitotic cells. HeLa cells were treated with siControl or siCep57 and immunostained with antibodies against PCNT (green) and ODF-2 (red). Scale bar, 200 nm. (**d**) Quantification of PCNT height on the centriole wall. Values are mean height ± s.d. from two independent experiments (siControl N=9, siCep57 N=10). Two-tailed, unpaired Student's t-test was used to obtain *P* value. *, $p < 0.05$; **, $p < 0.01$; NS, not significantly different ($p > 0.05$). (**e**) HeLa cells were treated with siControl or siCep57, followed by transfection with RNAi-resistant Cep57 or Cep57 mutant lacking the PCNT binding domain. The cells were immunostained with antibodies against HA (green), CP110 (red). Left-right arrows indicate precociously-disengaged centrioles. Scale bar, 5 μm in the low-magnified view, 1 μm in the inset. (**f**) BI2536 (Plk1 inhibitor) treatment blocked precocious centriole disengagement phenotype in Cep57-depleted cells. HeLa cells were treated with siControl or siCep57, followed by treatment with DMSO or BI2536 (500 nM) for 4 h. The cells were immunostained with antibodies against centrin-2 (green) and Cep192 (red). Scale bar, 5 μm in the low-magnified view, 1 μm in the inset. (**g**) Histograms represent frequency of mitotic cells with the indicated phenotypes in (f) (*N*=30 for each condition).

Vector	Primer name/sequence (forward)		Primer name/sequence (reverse)		Source
pCMV5_HA-Cep57	Cep57 1-_InsF	TTCCTGCAGgtcgacATGGCGGCGGCGTCTGTCTCTGC	Cep57 -500_InsR	CCGGGATCAggatccTCAGTAATCCCAACACAAACTACTGC	This study
pCMV5_HA-Cep57 RNAi-R	Cep57 RNAi-R_F	CTTAACTCCGACCTACGACGCTCCCCAAGTAAGCCTACAC	CEP57 RNAi-R_R	TAGGTCGGAGTTAAGGAAAGGCTTATCCGAAGGATATACTAC	This study
pCMV5_HA-Cep57 Δ68-103	Cep57Δ68-103_F	CAGCAGATATAAGAAAGTACTGGAT	Cep57Δ68-103_R	TTCTTATATCTGCTGTTGCTTTCTGG	This study
pCMV5_HA-Cep57 Δ120-160	Cep57Δ120-160_F	TTCAAAGAGGACATCTGTCTTAGAG	Cep57Δ120-160_R	GATGTCCTCTTTGAATTCTCCCTTTC	This study
pCMV5_HA-Cep57 Δ187-239	Cep57Δ187-239_F	CCAACTTACAAATAGACTTATCTTT	Cep57Δ187-239_R	CTATTTGTAAGTTGGCTCTGAACATG	This study
pCMV5_HA-Cep57 Δ68-103 RNAi-R	Cep57 RNAi-R_F	CTTAACTCCGACCTACGACGCTCCCCAAGTAAGCCTACAC	CEP57 RNAi-R_R	TAGGTCGGAGTTAAGGAAAGGCTTATCCGAAGGATATACTAC	This study
pCMV5_HA-Cep57 1-268	Cep57 1-_InsF	TTCCTGCAGgtcgacATGGCGGCGGCGTCTGTCTCTGC	Cep57 -268_InsR	CCGGGATCAggatcctcaTGGTGGTTTTGACTTCTTTTTTTTAATTCTTCTTGCATTGGG	This study
pCMV5_HA-Cep57 268-500	Cep57 268-_InsF	TTCCTGCAGgtcgacCCACCAGAAAAGAAAAGTTCTAGGAACTATTTTGGTGCAC	Cep57-500_InsR	CCGGGATCAggatccTCAGTAATCCCAACACAAACTACTGC	This study
pTB701_FLAG-PCNT					Ref. 1
pTB701_FLAG-PCNT RNAi-R	PCNT RNAi-R_F	GCAATcctgAGATTACGTTTTTTGGTT	PCNT RNAi-R_R	TAATCTcaggATTGCAATGACCACCCTG	This study
pTB701_FLAG-PCNT 1-1962	PCNT 1-_InsF	GAATTTTGCGGCCGCATGGAAGTTGAG	PCNT -1962_InsR	GCCATGACATTTTATtcaGGGCACCCGTGTGTGAGCTGTG	This study
pTB701_FLAG-PCNT 1962-3336	PCNT 1962-_InsF	GAATTTTGCGGCCGCCCCGGGGCCCACCCACAG	PCNT-3336_InsR	GCCATGACATTTTATTCACTGTTTAATCATCGGGTGG	This study
pTB701_FLAG-PCNT 2536-3336	PCNT 2536-_InsF	GAATTTTGCGGCCGCCAGGAGAAGCTGCAGCACTTGCGC	PCNT-3336_InsR	GCCATGACATTTTATTCACTGTTTAATCATCGGGTGG	This study
pTB701_FLAG-PCNT 2536-2944	PCNT 2536-_InsF	GAATTTTGCGGCCGCCAGGAGAAGCTGCAGCACTTGCGC	PCNT -2944_InsR	GCCATGACATTTTATtcaCTTCGACTCCAGGTCTCTCACTGTCTGC	This study
pTB701_FLAG-PCNT 2945-3336	PCNT 2945-_InsF	GAATTTTGCGGCCGCGACGAGGTGCCTGGCAGCCG	PCNT-3336_InsR	GCCATGACATTTTATTCACTGTTTAATCATCGGGTGG	This study
pTB701_FLAG-PCNT 2945-3138	PCNT 2945-_InsF	GAATTTTGCGGCCGCGACGAGGTGCCTGGCAGCCG	PCNT -3138_InsR	GCCATGACATTTTATtcaATGCAGGTACAATTTTTCCATCTTGACATTGCTGG	This study
pTB701_FLAG-PCNT 2945-3216	PCNT 2945-_InsF	GAATTTTGCGGCCGCGACGAGGTGCCTGGCAGCCG	PCNT -3216_InsR	GCCATGACATTTTATtcaCCATTTCTTAACCAAAAAACGTAATCTTAATATTGCAATGAC	This study
pTB701_FLAG-PCNT 3139-3336	PCNT 3139-_InsF	GAATTTTGCGGCCGCTACTTGAGAGCAGAGAGCTTTAGAAAAGCTCTG	PCNT-3336_InsR	GCCATGACATTTTATTCACTGTTTAATCATCGGGTGG	This study
pTB701_FLAG-PCNT 3217-3336	PCNT 3217-_InsF	GAATTTTGCGGCCGCCAAGAAGTAGATCGGAAAGGAGCTCTGGC	PCNT-3336_InsR	GCCATGACATTTTATTCACTGTTTAATCATCGGGTGG	This study
pTB701_FLAG-PCNT 2536-3336 Δ3139-3216 (ΔPACT)	PCNTΔ3139-3216_F	CCTGCATCAAGAAGTAGATCGGAAA	PCNTΔ3139-3216_R	ACTTCTTGATGCAGGTACAATTTTTC	This study
pTB701_FLAG-PCNT 2536-3336 R2918X	PCNT R2918X_F	GAAGGAGtGAGAATTAGAACTGCAGC	PCNT R2918X_R	AATTCTCaCTCCTTCTCTCTCCTGAG	This study
pTB701_FLAG-PCNT 2536-3336 K3154del	PCNTK3154del_F	TCAAAAGTATCTTTTGCTGTTGATT	PCNTK3154del_R	AAAAGATACTTTTGATAAATCAGAGC	This study
pTB701_FLAG-PCNT K3154del RNAi-R	PCNT RNAi-R_F	GCAATcctgAGATTACGTTTTTTGGTT	PCNT RNAi-R_R	TAATCTcaggATTGCAATGACCACCCTG	This study
pTB701_FLAG-PCNT Δ3139-3336-Cep57 chimera	PCNTΔPACT-Cep57_InsF	ggatccggtgcaggcgccGCGGCGGCGTCTGTCTCTGCG	PCNTΔPACT-Cep57_InsR	GCCATGACATTTTATTCAGTAATCCCAACACAAACTACTGCTTTGTAATG	This study
pGEX-6p-1_GST					GE Healthcare
pGEX-6p-1_GST-HA-Cep57 1-268	pGEX_HA-Cep57 1-_InsF	CTCAGATCTACTAGTatgGCGGCGGCGTCTGTCTCTGCG	pGEX_HA-Cep57 -268_InsR	CGCTCTAGAACTAGTtcaTGGTGGTTTTGACTTCTTTTTTTTAATTCTTCTTGCATTG	This study
pGEX-6p-1_GST-PCNT 3087-3336	pGEX_PCNT 3087-_InsF	GGGCCCCTGGGATCCGGCTGCAGCCCAAGCAGGTCG	pGEX_PCNT -3246_InsR	ATGCGGCCGCTCGAgtcaCTGTTTAATCATCGGGTGG	This study
pGEX-6p-1_GST-PCNT 3087-3336 Δ3139-3216 (ΔPACT)	PCNTΔ3139-3216_F	CCTGCATCAAGAAGTAGATCGGAAA	PCNTΔ3139-3216_R	ACTTCTTGATGCAGGTACAATTTTTC	This study
pGEX-6p-1_GST-AKAP9 3643-3907	pGEX_AKAP9 3643-_InsF	GGGCCCCTGGGATCCAACATTGAAGCCATCATTGCCTCTGAAAAAGAAG	pGEX_AKAP9 -3907_InsR	ATGCGGCCGCTCGAgttaTCTTCTCATGCCAGCATGAAATTGAGTAGTTGAAC	This study
pGEX-6p-1_MBP	pGEX_MBP_InsF	GAAACAGTATTCATGAAAATCGAAGAAGGTAAACTGGTAATCTGG	pGEX_MBP_InsR	AACTTCCAGATCCGAAGTCTGCGCGTCTTTCAGGGCTTC	This study
pGEX-6p-1_MBP-PCNT 3087-3336	pGEX_MBP_InsF	GAAACAGTATTCATGAAAATCGAAGAAGGTAAACTGGTAATCTGG	pGEX_MBP_InsR	AACTTCCAGATCCGAAGTCTGCGCGTCTTTCAGGGCTTC	This study
pSM378_Cep57 1-268	pSM378_Cep57 1-_InsF	GAATTCGGCGCGCCAatgGCGGCGGCGTCTGTCTC	pSM378_Cep57 -268_InsR	TCATGCGGCCGCTTAtcaTGGTGGTTTTGACTTCTTTTTTTTAATTCTTCTTGC	This study
pSM671_PCNT-3087-3336	pSM671_PCNT 3087-_InsF	GCTAGCGGCGCGCCAGGCTGCAGCCCAAGCAGGTCG	pSM671_PCNT -3336_InsR	GGTCGACGCGGCCGCTCACTGTTTAATCATCGGGTGGCAGGATTTC	This study
pSM671_PCNT-3087-3336 Δ3139-3216 (ΔPACT)	pSM671_PCNT 3087-_InsF	GCTAGCGGCGCGCCAGGCTGCAGCCCAAGCAGGTCG	pSM671_PCNT -3336_InsR	GGTCGACGCGGCCGCTCACTGTTTAATCATCGGGTGGCAGGATTTC	This study
pEGFP-C1					Addgene
pEGFP-C1_PCNT 3113-3336	GFP-PCNT 3113-_InsF	AATTCTGCAGTCGACGACCCCGGCCGGCTTCCAC	GFP-PCNT -3336_InsR	AGATCCGGTGGATCCtcaCTGTTTAATCATCGGGTGGCAGGATTTCTTTG	This study
pEGFP-C1_PCNT 3132-3226	GFP-PCNT 3132-_InsF	AATTCTGCAGTCGACATGGAAAAATTGTACCTGCATTACTTGAGAGCAG	GFP-PCNT -3226_InsR	AGATCCGGTGGATCCtcaTGCCAGAGCTCCTTTCCGATCTACTTCTTG	This study
pEGFP-C1_AKAP9 3643-3907	GFP-AKAP9 3643-_InsF	AATTCTGCAGTCGACAACATTGAAGCCATCATTGCCTCTGAAAAAGAAG	GFP-AKAP9 -3907_InsR	AGATCCGGTGGATCCttaTCTTCTCATGCCAGCATGAAATTGAGTAGTTGAAC	This study
pC1_HA-Cep152					Ref. 2
pEGFP-C1_Cep152					Ref. 2

Supplementary Figure 10. List of vectors and primers used in this study

Figure 4b

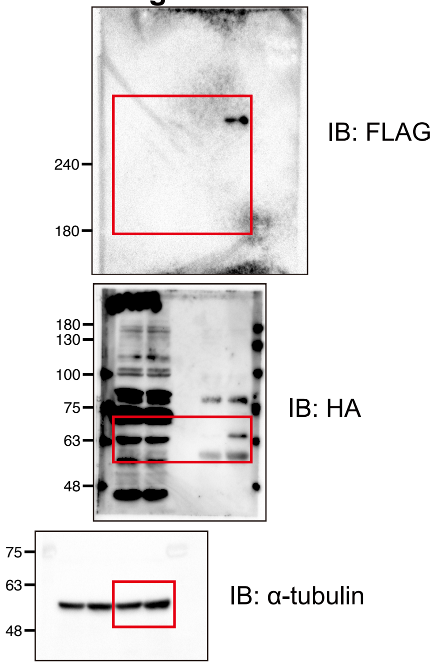

Figure 4c

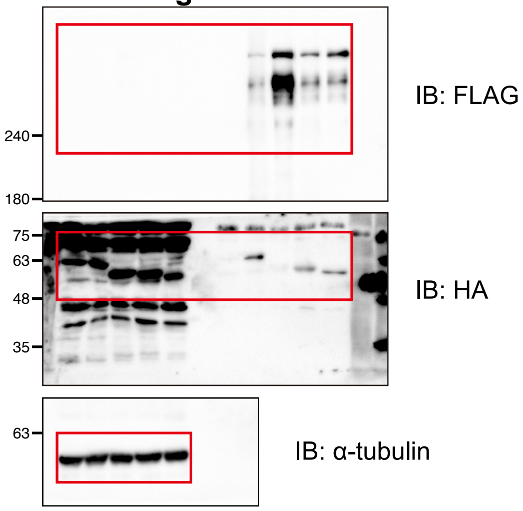

Figure 4e

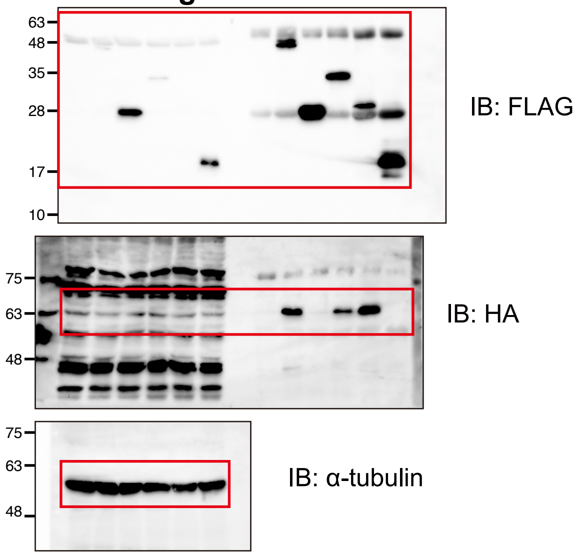

Figure 4f

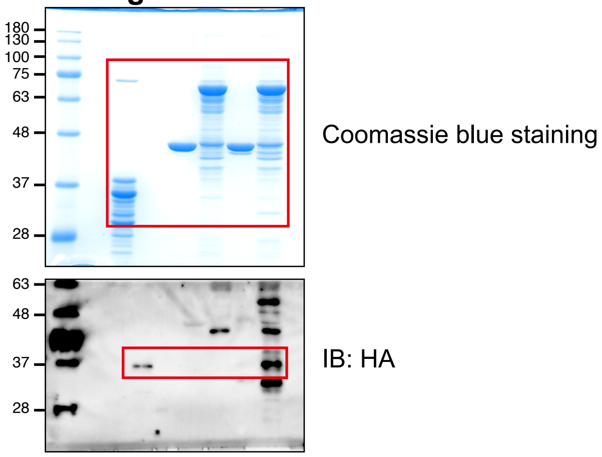

Figure 5b

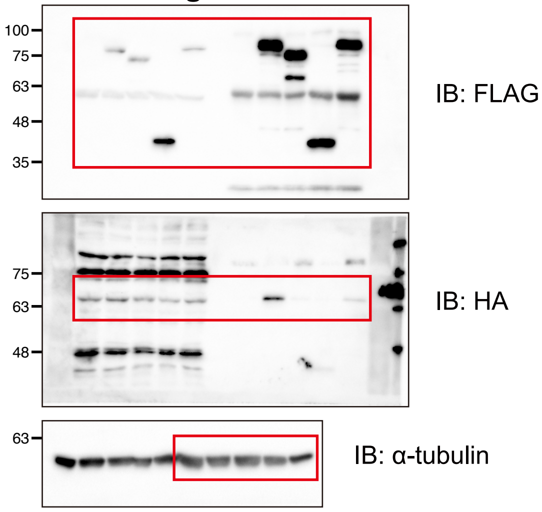

Supplementary Figure 11. Uncropped images of immunoblots and stained gels.

The cropped regions are indicated by red boxes. IB; immunoblot.

Supplementary References

1. Takahashi, M. *et al.* Centrosomal Proteins CG-NAP and Kendrin Provide Microtubule Nucleation Sites by Anchoring Γ-Tubulin Ring Complex. *Mol. Biol. Cell* **13,** 3235–3245 (2002).
2. Kim, T. S. *et al.* Hierarchical recruitment of Plk4 and regulation of centriole biogenesis by two centrosomal scaffolds, Cep192 and Cep152. *Proc. Natl. Acad. Sci.* (2013).
