# Supplementary material for: The Cep57-pericentrin module organizes PCM expansion and centriole engagement
Source: Nat Commun. 2019 Feb 25;10:931. doi: 10.1038/s41467-019-08862-2 (PMC6389942; doi:10.1038/s41467-019-08862-2)
Supplement: Supplementary file 3 — Description of Additional Supplementary Files [file 41467_2019_8862_MOESM3_ESM.pdf]

Descriptions of Additional Supplementary Information

File Name: Supplementary Movie 1.

Description: A control mitotic cell showing proper bipolar formation and chromosome segregation.

File Name: Supplementary Movie 2.

Description: A Cep57-depleted mitotic cell showing the precocious centriole disengagement phenotype.

File Name: Supplementary Movie 3.

Description: A Cep57-depleted mitotic cell showing unequal distribution of centrioles.

File Name: Supplementary Movie 4.

Description: A Cep57-depleted mitotic cell showing tripolar spindle formation.

File Name: Supplementary Movie 5.

Description: A Cep57-depleted mitotic cell showing chromosome misalignment and multi-nuclei.

File Name: Supplementary Movie 6.

Description: A Cep57-depleted mitotic cell showing lagging chromosome.
