# Supplementary material for: The Cep57-pericentrin module organizes PCM expansion and centriole engagement
Source: Nat Commun. 2019 Feb 25;10:931. doi: 10.1038/s41467-019-08862-2 (PMC6389942; doi:10.1038/s41467-019-08862-2)
Supplement: Supplementary file 10 — Reporting Summary [file 41467_2019_8862_MOESM10_ESM.pdf]

nature research

Corresponding author(s): Daiju Kitagawa

Last updated by author(s): Dec 25, 2018

Reporting Summary

Nature Research wishes to improve the reproducibility of the work that we publish. This form provides structure for consistency and transparency in reporting. For further information on Nature Research policies, see Authors & Referees and the Editorial Policy Checklist.

Statistics

For all statistical analyses, confirm that the following items are present in the figure legend, table legend, main text, or Methods section.

n/a	Confirmed	
☐	☒	The exact sample size (*n*) for each experimental group/condition, given as a discrete number and unit of measurement
☐	☒	A statement on whether measurements were taken from distinct samples or whether the same sample was measured repeatedly
☐	☒	The statistical test(s) used AND whether they are one- or two-sided<br>*Only common tests should be described solely by name; describe more complex techniques in the Methods section.*
☐	☒	A description of all covariates tested
☐	☒	A description of any assumptions or corrections, such as tests of normality and adjustment for multiple comparisons
☐	☒	A full description of the statistical parameters including central tendency (e.g. means) or other basic estimates (e.g. regression coefficient) AND variation (e.g. standard deviation) or associated estimates of uncertainty (e.g. confidence intervals)
☐	☒	For null hypothesis testing, the test statistic (e.g. *F*, *t*, *r*) with confidence intervals, effect sizes, degrees of freedom and *P* value noted<br>*Give P values as exact values whenever suitable.*
☒	☐	For Bayesian analysis, information on the choice of priors and Markov chain Monte Carlo settings
☒	☐	For hierarchical and complex designs, identification of the appropriate level for tests and full reporting of outcomes
☒	☐	Estimates of effect sizes (e.g. Cohen's *d*, Pearson's *r*), indicating how they were calculated

Our web collection on statistics for biologists contains articles on many of the points above.

Software and code

Policy information about availability of computer code

Data collection	Images were collected using SoftWoRx (Applied Precision) and LAS X (Leica).
Data analysis	Images were analyzed by FIJI distribution of ImageJ. Statistical analyses were performed with GraphPad Prism 7. For deconvolution, Huygens essential software (SVI; Scientific Volume Imaging) was used.

For manuscripts utilizing custom algorithms or software that are central to the research but not yet described in published literature, software must be made available to editors/reviewers. We strongly encourage code deposition in a community repository (e.g. GitHub). See the Nature Research guidelines for submitting code & software for further information.

Data

Policy information about availability of data

All manuscripts must include a data availability statement. This statement should provide the following information, where applicable:

- Accession codes, unique identifiers, or web links for publicly available datasets
- A list of figures that have associated raw data
- A description of any restrictions on data availability

The data that support the findings of this study are available from the corresponding author upon request.

Field-specific reporting

Please select the one below that is the best fit for your research. If you are not sure, read the appropriate sections before making your selection.

☒ Life sciences ☐ Behavioural & social sciences ☐ Ecological, evolutionary & environmental sciences

October 2018

Life sciences study design

All studies must disclose on these points even when the disclosure is negative.

Sample size	No statistical method was used to predetermine sample size.
Data exclusions	No data were excluded from the analyses.
Replication	Reproducibility was confirmed. The number of experiments is described in the figure legends.
Randomization	We assessed cells from several fields for each experiment. The field were chosen randomly. Once a field was determined, we counted all cells which match the criteria within the filed.
Blinding	The investigators were not blinded to the sample ID during experiments and outcome assessment because the mitotic cells in randomly selected area were analysed objectively.

Reporting for specific materials, systems and methods

We require information from authors about some types of materials, experimental systems and methods used in many studies. Here, indicate whether each material, system or method listed is relevant to your study. If you are not sure if a list item applies to your research, read the appropriate section before selecting a response.

Materials & experimental systems

n/a	Involved in the study	
☐	☒	Antibodies
☐	☒	Eukaryotic cell lines
☒	☐	Palaeontology
☒	☐	Animals and other organisms
☒	☐	Human research participants
☒	☐	Clinical data

Methods

n/a	Involved in the study	
☒	☐	ChIP-seq
☒	☐	Flow cytometry
☒	☐	MRI-based neuroimaging

Antibodies

Antibodies used	The following primary antibodies were used in this study: rabbit antibodies against Cep57 (GeneTex, GTX115931, IF 1:1000), PCNT (Abcam, ab4448, IF 1:2000), Cep192 (Bethyl Laboratories, A302–324A, IF 1:1000), Cep152 (Bethyl Laboratories, A302–480A, IF 1:1000), CP110 (Proteintech, 12780–1-AP, IF 1:500), CDK5RAP2 (Bethyl Laboratories, IHC-0063, IF 1:1000), Mad1 (Bethyl Laboratories, A300–339A, IF 1:500), ODF-2 (Abcam, ab43840, IF 1:1000) and HA-tag (Abcam, ab9110, IF 1:1000, WB 1:1000); mouse antibodies against Cep57 (Abcam, ab169301, IF1:1000), PCNT (Abcam, ab28144, IF 1:2000), centrin-2 (Merck, 20H5, IF 1:1000), γ-tubulin (GTU88) (Merck, T5192, IF 1:1000), Polyglutamylation modification (GT335) (AdipoGen, AG-20B-0020-C100, IF 1:1000), GFP (MBL, 598 , IF 1:1000, WB 1:1000), FLAG-tag (Merck, F1804, IF 1:1000, WB 1:1000) and α-tubulin (Merck, DM1A, IF1:1000, WB 1:1000) ; goat antibodies against PCNT (Santa Cruz Biotechnology, sc-218145, 1:300); guinea pig antibodies against CENP-C (MBL, PD030, IF 1:1000); Alexa 488-labelled Cep152 (Bethyl Laboratories, A302–480A, IF 1:500) and ODF-2 (Abcam, ab43840, IF 1:100) and Alexa 647-labelled Cep192 (Bethyl Laboratories, A302–324A, IF 1:500) were generated with Alexa Fluor labelling kits (Life Technologies). The following secondary antibodies were used: Alexa Fluor 488 goat anti-mouse IgG (H+L) (Molecular Probes, A-11001, 1:1000), Alexa Fluor 568 goat anti-rabbit IgG (H+L) (Molecular Probes, A-11011, 1:1000), Alexa Fluor 568 goat anti-guinea pig IgG (H+L) (Abcam, ab175714, 1:1000), Alexa Fluor 555 donkey anti-rabbit IgG (H+L) (Abcam, ab150074, 1:1000) and Alexa Fluor 488 donkey anti-goat IgG (H+L) (Abcam, ab150129, 1:1000) for IF; Alexa Fluor 555 goat anti-rabbit IgG (H+L) (Molecular Probes, A-21428, 1:500) for STED; Goat polyclonal antibodies-horseradish peroxidase against mouse IgG (Promega, W402B, 1:5000) and rabbit IgG (Promega, W401B, 1:5000) for WB.
Validation	Method section provided with manuscript contains information on all antibodies used in the study. The validation of each primary antibody was performed by immunofluorescence staining.

Eukaryotic cell lines

Policy information about cell lines

Cell line source(s)	HeLa, U2OS, RPE-1 and HEK293T cells were obtained from the ECACC (European collection of cell cultures). 293FT cells were obtained from ThermoFisher.
Authentication	HeLa, U2OS, RPE-1 and HEK293T cells have been authenticated by STR profiling in ECACC.
Mycoplasma contamination	It have been confirmed that HeLa and U2OS were not contaminated with mycoplasma by indirect DNA stain using Hoechst

Mycoplasma contamination	33258 with indicator cells (Vero cells). It has been confirmed that RPE-1, HEK293T and 293FT were not contaminated with mycoplasma in ECACC or ThermoFisher before providing.
Commonly misidentified lines<br>(See ICLAC register)	Cells used in this study are not listed in the database of commonly misidentified cell lines maintained by ICLAC.
